# The impact of pre-existing aortic stenosis and mitral regurgitation on patients with acute myocardial infarction

**DOI:** 10.1038/s41598-025-01313-7

**Published:** 2025-05-20

**Authors:** Tamilla Muzafarova, Zuzana Motovska, Petr Kala, Ota Hlinomaz, Milan Hromadka, Teodora Vichova, Jan Mrozek, Marek Šramko, Martin Hutyra, Robert Petr, Pavol Tomasov, Oana Ionita, Pilar Lopez Santi, Aileen Paula Chua, Jiri Jarkovsky

**Affiliations:** 1 Cardiocentre, Third Faculty of Medicine, Charles University and University Hospital Kralovske Vinohrady, Srobarova 50, Prague, 10034 Czech Republic; 2 Department of Internal Medicine and Cardiology, Faculty of Medicine of Masaryk University and University Hospital Brno, Brno, Czech Republic; 3 First Department of Internal Medicine - Cardioangiology ICRC Faculty of Medicine of Masaryk, University and St. Anne’s University Hospital, Brno, Czech Republic; 4 Department of Cardiology, Faculty of Medicine in Pilsen, University Hospital, Charles University, Pilsen, Czech Republic; 5 Cardiovascular Department University Hospital Ostrava, Ostrava, Czech Republic; 6 Department of Cardiology Institute for Clinical and Experimental Medicine, Olomouc, Czech Republic; 7 First Internal, Cardiology Clinic University Hospital Olomouc, Olomouc, Czech Republic; 8 Cardiology Prague Ltd, Prague, Czech Republic; 9 Liberec Regional Hospital, Liberec, Czech Republic; 10 Leiden University Medical Centre, Leiden, Netherlands; 11 Institute of Biostatistics and Analyses Faculty of Medicine, Masaryk University, Brno, Czech Republic; 12 The Institute of Health Information and Statistics of the Czech Republic, Prague, Czech Republic

**Keywords:** Cardiovascular biology, Risk factors

## Abstract

The prevalence of left-sided valvular heart disease (VHD) increases with age, but data on the impact of pre-existing VHD in patients with acute myocardial infarction (AMI) are limited. We aimed to define the clinical characteristics and outcomes of AMI patients with pre-existing left VHD. The analysis is based on data from three merged national registries. The dataset included 47,436 patients admitted with AMI over a 5year period at all Cath Labs nationwide. Pre-existing VHD was diagnosed in 1,445 patients (3.0%), moderate-to-severe mitral regurgitation (MR) in 510 patients (35.3%), and moderate-to-severe aortic stenosis (AS) in 869 patients (60.1%). Patients with VHD had worse baseline characteristics, pre-existing coronary artery disease, more complicated in-hospital course with higher Killip class, lower left ventricular ejection fraction, and more comorbidities. Angiographically more frequent left main stenosis, TIMI flow 3 before PCI, less frequent stent implantation. Patients with pre-existing VHD had significantly higher 7-day (10.1% vs. 4.5%, *p* < 0.001), 30-day (16.0% vs. 7.0%, *p* < 0.001) and 1-year mortality (28.7 vs. 12.7%, *p* < 0.001) compared to patients without. Conclusions. Patients with pre-existing VHD and AMI are characterized by complicated in-hospital course with higher Killip class, lower ejection fraction, angiographically less severe stenosis, TIMI flow 3 prior to PCI, and less frequent stent implantation. This is a high-risk group with higher short – and long-term mortality and earlier intervention should be considered.

## Introduction

Acute myocardial infarction (AMI) remains the leading cause of mortality and morbidity worldwide, despite substantial improvements in management strategies over the past decades^1^Aortic stenosis (AS) and mitral regurgitation (MR) are the most common acquired valvular heart disease (VHD) in developed countries^2^, and their prevalence is expected to nearly double by 2030^3^ The strong association between coronary artery disease (CAD) and VHD has been demonstrated in a number of large population-based studies^4–7^ The left-sided VHD and CAD share common risk factors (smoking, dyslipidemia, diabetes and arterial hypertension) and underlying pathophysiological mechanisms (endothelial dysfunction, lipid deposition, inflammation)^8,9^ Pre-existing VHD is more common in AMI patients than in the general population, with AS ranging from 2.7 to 16% and MR from 2.4 to 13.2% in AMI patients^10,11^ VHD and AMI mutually promote a series of pathophysiological changes that lead to progression of VHD and worse outcome in AMI. Cardiac remodeling in AMI, left ventricular hypertrophy with increased myocardial oxygen demand, elevated interventricular pressure, microvascular compression, arteriolar remodeling and fibrosis, limit adequate coronary perfusion, resulting in an oxygen demand/supply mismatch and ischemia^12^ In addition, AMI affects valvular changes with increased collagen production, thickening and remodeling of the aortic and mitral valves, leading to progression of AS and MR^13,14^ With population growth, aging and increasing prevalence of cardiovascular risk factors, the global number of patients with AMI and VHD is expected to increase substantially^2,3^ This analysis aims to investigate the prevalence and prognostic impact of pre-existing left VHD in patients with acute myocardial infarction.

## Methods

### Study population

Patients who underwent cardiac catheterization for AMI in the Czech Republic between 1 January 2017 and 31 December 2021 were included. Patients who underwent cardiac catheterization for diagnoses other than AMI were not included in the study. Demographic and clinical data at baseline were collected from the prospective national multicenter registry, which collects data on all cardiac catheterizations performed in the country. The medical reports contain demographic, clinical, electrocardiographic and angiographic characteristics, including complications. Enrolled patients with myocardial infarction were screened for the presence/absence of pre-existing significant left VHD, defined as moderate-to-severe and severe AS or MR. Data were collected from the National Registry of Reimbursed Health Services using ICD-10 CM codes - I35.0, I35.2 for AS and I34.0 for MR in one of the secondary diagnosis fields. The study endpoint was 30-day all-cause mortality. Mortality data were based on the National Death Registry.

### Statistical analysis

Standard descriptive statistics were used in the analysis: absolute and relative frequencies for categorical variables and means with standard deviations for continuous variables. The statistical significance of differences between patient groups was tested using the maximum likelihood chi-squared test for categorical variables and the t-test for continuous variables. The Kaplan-Meier method was used to visualize the overall survival data; the Cox proportional hazards model was used to analyze the association between VHD and overall patient survival and to describe it using hazard ratios and their 95% confidence intervals. Overall survival was analyzed both since catheterization and after 30 days using the landmark analysis approach. The association between VHD, patient characteristics and the endpoint of 30-day all-cause mortality was analyzed using logistic regression and adjusted for potential confounders in the multivariate model. Analyses were performed using SPSS software (version 28.0.1.1.), and *p* = 0.05 was used as the level of statistical significance in all analyses.

## Results

### Prevalence and characteristics

A total of 48,881 patients with AMI were enrolled during the 5-year study period (2017–2021) in the Czech Republic. The 1,445 patients (3%) with significant AS or MR were assigned to the “VHD” group. The predominant valvular disease in the VHD group was AS, which was observed in 869 patients (60.1%). Within each group, the demographic, clinical and angiographic characteristics of the patients and their outcomes were compared with those of the non-VHD patients. Baseline characteristics are shown in **Table** 1. Based on information on electrocardiographic patterns, non-ST-elevation MI was a more frequent manifestation in the VHD group. Patients in the VHD group were older and more likely to be female. The prevalence of both AS and MR increased with age. Patients in the VHD group were also more likely to have diabetes, chronic renal failure, a history of previous ischemic heart disease or revascularization. On admission, VHD patients had a higher prevalence of mild to moderate heart failure and pulmonary edema (Killip > 2), and a higher incidence of left ventricular dysfunction with a higher proportion of left ventricular ejection fraction (LVEF) < 30%, compared to the non-VHD group. In terms of coronary intervention, the VHD group included a greater extent of coronary artery disease with multivessel and left main disease. The AMI patients with VHD had a higher proportion of Thrombolysis in Myocardial Infarction (TIMI) flow 3 prior to percutaneous coronary intervention and more frequent ≤ 80% stenosis in the infarct-related coronary artery. Among the patients with VHD, the presence of AS was independently associated with more severe heart failure (Killip 3 and 4) and a greater use of mechanical ventilation. **(Table** 2)

**Table 1:** Baseline and angiographic characteristics.

		Non-VHD	VHD	*p* ^1^	MR	*p* ^1^	AS	*p* ^1^	*p* ^2^
		47,436	1,445		510		869		
Female gender, %		28.9	39.6	*< 0.01*	49.2	*< 0.01*	33.6	*< 0.01*	*< 0.01*
Age, mean (± SD), yrs.		65.6 (± 12.5)	73.9 (± 10.6)	*< 0.01*	72.4 (± 11.1)	*< 0.01*	74.7 (± 10.1)	*< 0.01*	*< 0.01*
Age, yrs. %	< 40	1.8	0.6	*< 0.01*	0.6	*0.04*	0.7	*< 0.01*	*1.00*
	40–49	9.7	2.9	*< 0.01*	5.5	*< 0.01*	1.4	*< 0.01*	*< 0.01*
	50–59	19.4	5.6	*< 0.01*	5.5	*< 0.01*	5.8	*< 0.01*	*0.91*
	60–69	28.9	18.8	*< 0.01*	20.6	*< 0.01*	17.6	*< 0.01*	*0.18*
	70–79	26.2	40.6	*< 0.01*	40.6	*< 0.01*	40.6	*< 0.01*	*1.00*
	≥ 80	14.0	31.5	*< 0.01*	27.3	*< 0.01*	33.9	*< 0.01*	*0.01*
AMI type, %	STEMI	57.7	41.2	*< 0.01*	44.1	*< 0.01*	39.1	*< 0.01*	*0.12*
	NSTEMI	41.3	58.9	*< 0.01*	55.9	*< 0.01*	60.9	*< 0.01*	*0.07*
Diabetes mellitus, %		20.0	27.6	*< 0.01*	22.7	*0.13*	30.1	*< 0.01*	*< 0.01*
Chronic kidney disease, %		5.0	11.9	*< 0.01*	13.5	*< 0.01*	11.2	*< 0.01*	*0.20*
Previous PCI, %		14.9	23.5	*< 0.10*	22.2	*< 0.01*	24.1	*< 0.01*	*0.43*
Previous CABG, %		5.3	23.4	*< 0.01*	16.9	*< 0.01*	26.4	*< 0.01*	*< 0.01*
Killip class,%	Killip 1	81.4	73.4	*< 0.01*	76.5	*< 0.01*	71	*< 0.01*	*0.03*
	Killip 2	7.7	11.6	*< 0.01*	10.8	*0.01*	12.4	*< 0.01*	*0.40*
	Killip 3	3.5	7.3	*< 0.01*	5.5	*0.02*	8.4	*< 0.01*	*0.05*
	Killip 4	5.7	5.9	*0.69*	4.3	*0.21*	6.9	*0.12*	*0.05*
	missing	1.7	1.8	*-*	2.9	*-*	1.3	*-*	*-*
OHCA, %		8.5	8.4	*0.84*	8.6	*0.95*	8.1	*0.66*	*0.76*
Mechanical ventilation, %		9.0	9.9	*0.24*	7.8	*0.39*	11.3	*0.02*	*0.04*
LVEF*, %	>50%	49.6	44.5	*< 0.01*	42.3	*0.02*	45.9	*0.11*	*0.33*
	30–50%	41.4	43.0	*0.35*	43.0	*0.58*	42.4	*0.65*	*0.88*
	<30%	9.0	12.5	*< 0.01*	14.7	*< 0.01*	11.7	*0.05*	*0.26*
Number of diseased vessels, %	1-VD	45.1	37.2	*< 0.01*	37.8	*< 0.01*	37.2	*< 0.01*	*0.82*
	2-VD	29.6	28.3	*0.28*	28.8	*0.73*	28.9	*0.65*	*1.00*
	3-VD	22.6	30.6	*< 0.01*	28.6	*< 0.01*	30.4	*< 0.01*	*0.50*
Infarct related coronary artery, %	LMCA	4.0	8.4	*< 0.01*	4.9	*0.31*	10.4	*< 0.01*	*< 0.01*
	RIA	44.8	44.2	*0.67*	44.1	*0.79*	44.3	*0.78*	*0.96*
	RCx	25.0	26.0	*0.39*	23.3	*0.41*	27.5	*0.10*	*0.10*
	ACD	35.2	30.9	*< 0.01*	34.3	*0.71*	29.1	*< 0.01*	*0.05*
Stenosis before PCI, %	≤ 80%	13.1	18.8	*< 0.01*	16.3	*0.04*	20.0	*< 0.01*	*0*,*09*
	81–90%	21.5	25.9	*< 0.01*	25.5	*0.03*	26.7	*< 0.01*	*0*,*66*
	91–99%	26.7	27.0	*0.79*	27.5	*0.69*	26.0	*0.70*	*0*,*57*
	100,0%	38.8	28.4	*< 0.01*	30.8	*< 0.01*	27.3	*< 0.01*	*0*,*17*
TIMI flow before PCI, %	0	37.8	27.2	*< 0.01*	29.8	*< 0.01*	26.1	*< 0.01*	*0.15*
	1	7.6	8.9	*0.09*	8.6	*0.41*	8.9	*0.18*	*0.92*
	2	16.6	16.9	*0.75*	17.8	*0.44*	16.2	*0.81*	*0.46*
	3	38	47.1	*< 0.01*	43.7	*< 0.01*	48.8	*< 0.01*	*0.07*
TIMI flow after PCI, %	< 3	8.2	9.3	*0.11*	10.4	*0.07*	8.4	*0.81*	*0.25*
	3	91.8	90.7	*0.11*	89.6	*0.07*	91.6	*0.81*	*0.25*
IABK		0.0	0.0	*1.00*	0.0	*1.00*	0.0	*1.00*	*-*
ECMO		0.3	0.3	*0.79*	0.0	*0.65*	0.5	*0.29*	*0.30*
Middle term mech. support		0.1	0.1	*1.00*	0.0	*1.00*	0.1	*0.55*	*1.00*
Long term mech. support		0.1	0.1	*0.57*	0.0	*1.00*	0.1	*0.40*	*1.00*
MitraClip		0.1	0.1	*1.00*	0.2	*0.39*	0.0	*1.00*	*0.37*
TAVI		14.0	20.8	*< 0.01*	16.5	*0.11*	23.2	*< 0.01*	*< 0.01*

ACD, arteria coronaria dextra; AS, aortic stenosis; CABG, coronary artery bypass grafting; CKD, chronic kidney disease; CPR, cardiopulmonary resuscitation; LMC, left main coronary artery; LVEF, left ventricular ejection fraction; MR, mitral regurgitation; NSTEMI, non-ST elevation myocardial infarction; OHCA, out of hospital cardiac arrest; PCI, percutaneous coronary intervention; RIA, ramus interventricularis anterior; RCx, ramus circumflexus; SD, standard deviation; STEMI, ST-elevation myocardial infarction; TIMI, thrombolysis in myocardial infarction; VD, vessel disease; VHD, valvular heart disease;.

* Information on LVEF is available in 53–58% of patients, results are computed only for patients with available data.

* p1 represents the comparison between non-VHD and each given VHD type, p2 represents the comparison between MR and AS.

**Table 2:** Valvular heart disease as risk factors of mechanical ventilation and heart failure in MI patients.

		Mechanical ventilation		Killip > 2		Killip 3–4	
Predictor	N	OR (95%CI)	*p*	OR (95% CI)	*p*	OR (95% CI)	*p*
NonVHD	47.436	reference					
MR	510	0.86 (0.62;1.19)	*0.37*	1.30 (1.04;1.61)	*0.02*	1.09 (0.81;1.46)	*0.56*
AS	869	1.29 (1.04;1.59)	***0.02***	1.88 (1.62;2.19)	***< 0.01***	1.78 (1.48;2.15)	***< 0.01***

AS, aortic stenosis; MR, mitral regurgitation; VHD, valvular heart disease.

**Table 3:** Multivariate model for short- and long-term all-cause mortality of MI patients.

Factor		30 days		Long-term	
		OR (95%CI)	*p*	OR (95%CI)	*p*
**MR**		**1.01 (0.82–1.46)**	***0.53***	**1.21 (1.04–1.40)**	***0.01***
**AS**		**1.80 (1.51–2.13)**	***< 0.01***	**1.63 (1.47–1.81)**	***< 0.01***
Age		1.04 (1.04–1.05)	*< 0.01*	1.05 (1.05–1.05)	*< 0.01*
Woman		1.01 (1.02–1.18)	*0.01*	1.06 (1.02–1.11)	*< 0.01*
STEMI		1.44 (1.33–1.56)	*< 0.01*		
Diabetes mellitus		1.23 (1.13–1.32)	*< 0.01*	1.29 (1.23–1.35)	*< 0.01*
Chronic kidney disease		1.04 (0.93–1.17)	*< 0.01*	1.35 (1.27-1,44)	*< 0.01*
Previous PCI				1.05 (1.01–1.10)	*0.05*
Previous CABG		0.84 (0.73–0.96)	*< 0.01*	1.13 (1.05–1.21)	*< 0.001*
Killip class	Killip 1	*Reference*			
	Killip 2	0.55 (0.40–0.75)	*< 0.01*	1.72 (1.62–1.82)	*< 0.01*
	Kilip 3	1.41 (1.02–1.93)	*0.04*	2.05 (1.90–2.22)	*< 0.01*
	Kilip 4	1.71 (1.23–2.36)	*0.01*	3.74 (3.48–4.03)	*< 0.01*
OHCA		2.12 (1.85–2.45)	*< 0.01*	1.65 (1.53–1.78)	*< 0.01*
Mechanical ventilation		1.93 (1.76–2.12)	*< 0.01*	1.13 (1.04–1.22)	*< 0.01*
No. of diseased vessels	1 VD	*Reference*			
	2 VD	1.06 (0.97–1.16)	*0.21*	1.10 (1.05–1.16)	*< 0.01*
	3 VD	1.24 (1.14–1.36)	*< 0.01*	1.30 (1.23–1.36)	*< 0.01*
LVEF	> 50%	*Reference*			
	30–50%	1.81 (1.57–2.08)	*< 0.01*	1.48 (1.40–1.57)	*< 0.01*
	< 30%	3.22 (2.77–3.75)	*< 0.01*	2.40 (2.22–2.59)	*< 0.01*
LMC stenosis > 50%		1.35 (1.22–1.48)	*< 0.01*	1.20 (1.13–1.28)	*< 0.01*

AS, aortic stenosis; CABG, coronary artery bypass grafting; CPR, cardiopulmonary resuscitation; LMC, left main coronary artery; MI, myocardial infarction; MR, mitral regurgitation; TIMI, thrombolysis in myocardial infarction; VD, vessel disease; VHD, valvular heart disease.

### Short- and long-term outcome

**Fig. 1:**
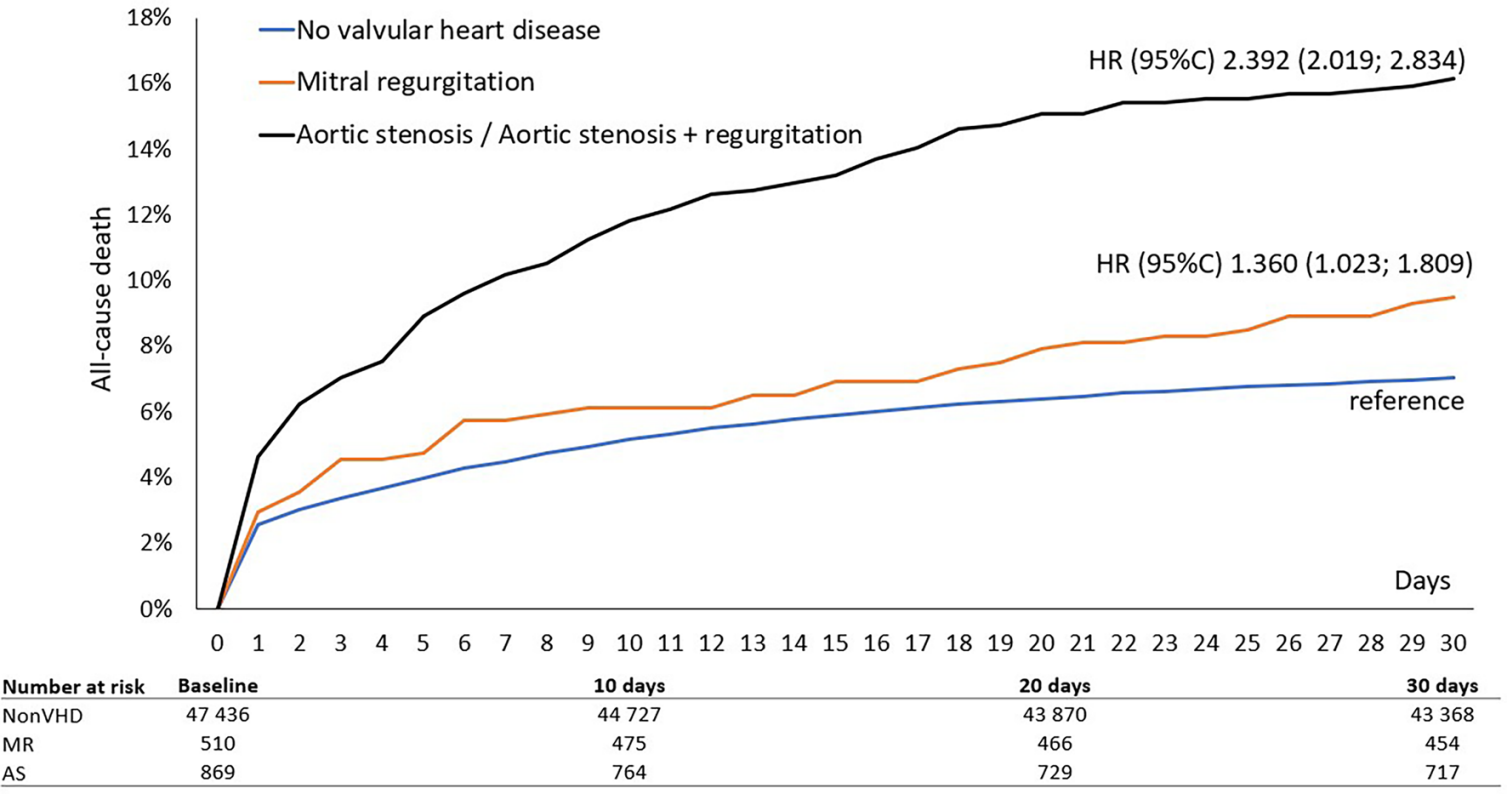
Thirty days survival according to valvular heart disease.

**Fig. 2:**
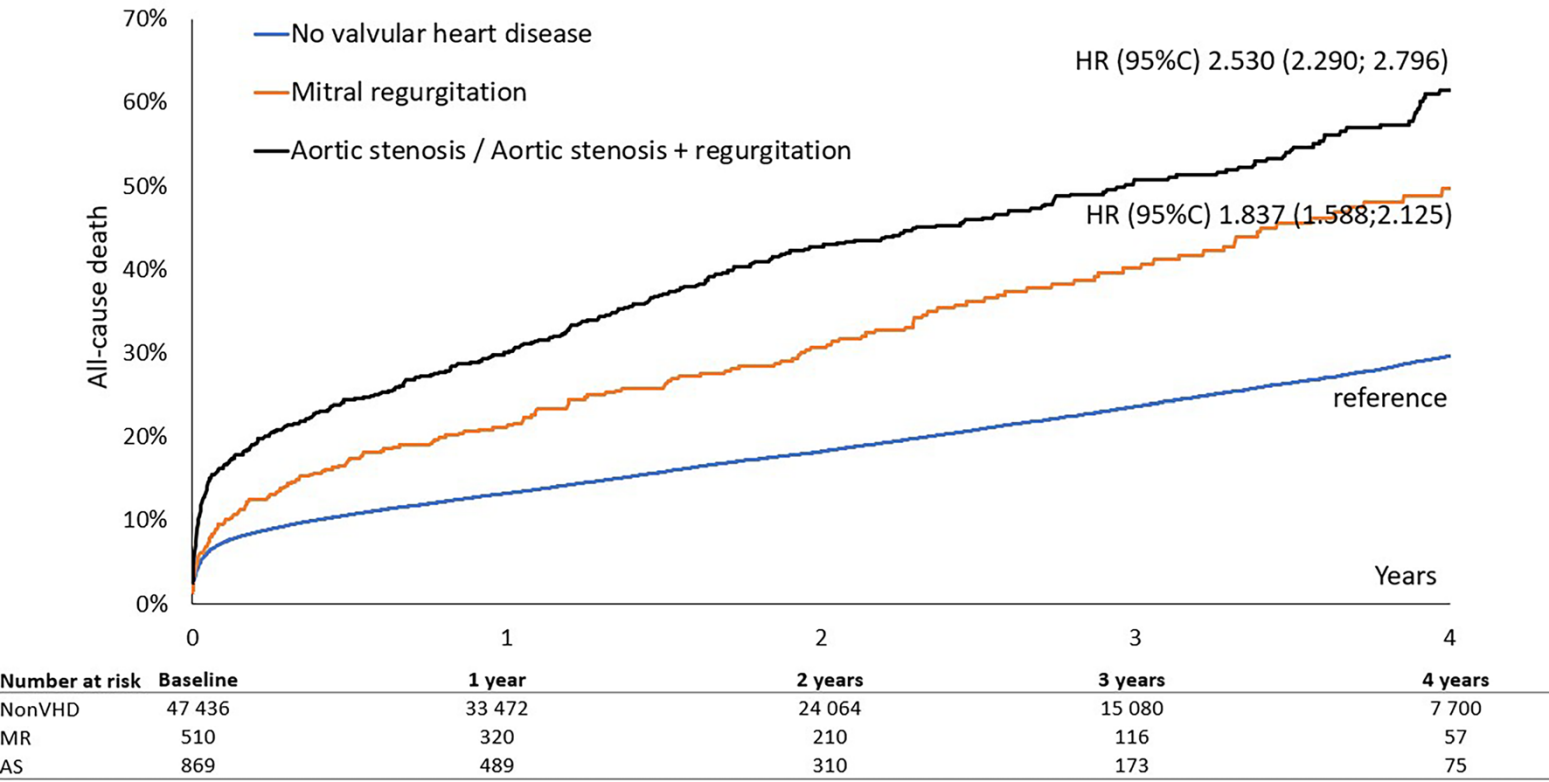
**L**ong-term survival according to valvular heart disease.

**Fig. 3:**
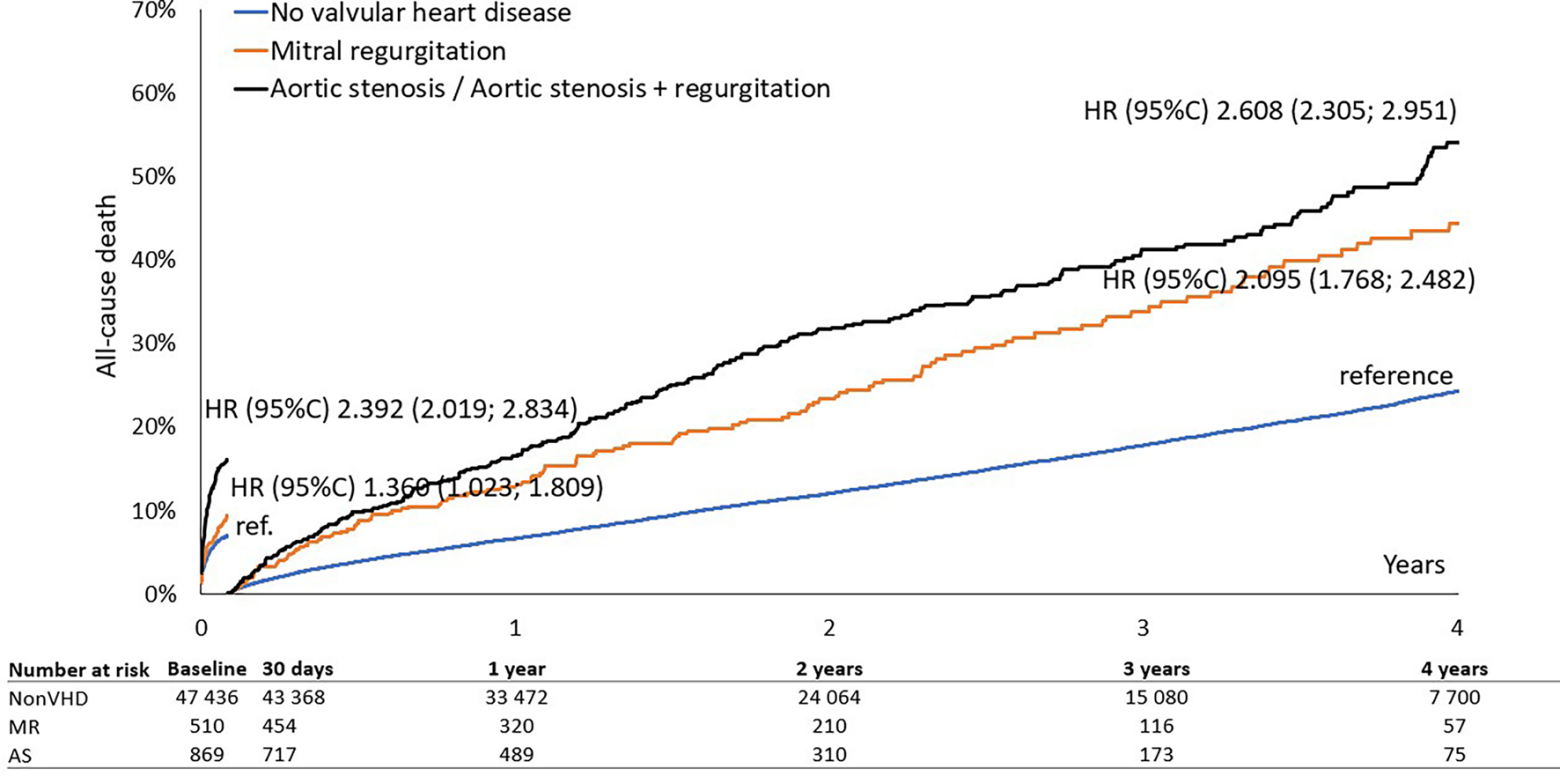
Survival until 30 days and long-term survival since 30 days according to valvular heart disease – landmark analysis.

In-hospital and 30-day all-cause mortality was nearly double in patients with VHD compared to those without, 8.3% vs. 4.5% and 13.4% vs. 7%, respectively, *p* < 0.01 for all. In the VHD group, patients with significant AS had a higher in-hospital and 30-day mortality rates than did patients with significant MR, 10.1% vs. 5.7% and 16.0% vs. 9.4%, respectively, *p* < 0.01 for all. The adjusted risk for patients with pre-existing AS was high for both short- and long-term mortality (odds ratio (OR) 1.80; 95% confidence interval (CI) 1.51–2.13) and (OR 1.63; 95% CI 1.47–1.81), respectively *p* < 0.01 for all. **(Table **3**)** In multivariate analysis, MR did not significantly increase the risk of 30-day mortality after adjustment for covariates and potential confounders, but remained independently associated with long-term all-cause mortality (OR 1.21; 95% CI 1.04–1.40), *p* < 0.01. **(Table **3**)** The Kaplan-Meier survival curves showed early divergence of the curves among the groups with AS, MR and non-VHD, with significantly higher 30-day mortality in AS, followed by MR and non-VHD **(Fig. **1**.).** The difference between the AS and MR survival curves becomes smaller in a long-term period **(Fig. **2**)** and the landmark analysis after 30 days demonstrates the later divergence of AS and MR curves **(Fig. **3**).**

## Discussion

The vast majority of studies report about valvular complications after AMI. We have analyzed patients with pre-existing significant VHD and have described that they are not uncommon in AMI patients, with a prevalence of about 3% and a tendency to increase with age. Published studies report VHD in 4.8% of ACS patients^10^, rising up to approximately 20% of ACS patients in the elderly population > 74 years of age^11^Unlike the aforementioned studies in selected population^10,11^, our study analyzed a nationwide all-comers registry of AMI patients, which may explain the differences in prevalence. With the population growth, aging and increasing cardiovascular risk factors, pre-existing VHD in AMI is a rising problem.

The presence of VHD in AMI is associated with more severe course of the disease and outcome. According to our data, these patients are older, have a higher risk profile, have more comorbidities, they more often present with mild to moderate heart failure and pulmonary edema, with a lower LVEF, with a wider extent of coronary artery disease with multivessel and left main disease, they have more complicated in-hospital course with advanced stages of heart failure including cardiogenic shock and acute multiorgan failure, which is consistent with other published data^10,11,15^ Both AS and MR increase the workload on the left ventricle, lead to cardiac remodeling, and contribute to pulmonary congestion and dyspnea. In the inpatient setting, these conditions can progress rapidly to acute decompensated heart failure, requiring intensive management and possibly surgical intervention to prevent further deterioration. The presence of pre-existing significant VHD adversely affects the prognosis of AMI patients, leading to an almost twofold increase in in-hospital and 30-day mortality rate compared to AMI patients without VHD. The higher risk of cardiovascular mortality, three times higher risk of composite of all-cause death, AMI, stroke and rehospitalization for heart failure was reported for AMI patients with VHD in the study by Crimi et al.^11^ According to our analysis the presence of AS is independently associated with worse prognosis and have higher short- and long-term mortality compared to the AMI patients with MR or AMI patients without VHD. As in Kaplan-Meier analysis, the mortality curve of AS separated early in the 30-day analysis and had a later divergence with MR curve in landmark analysis after 30 days, suggesting that the presence of AS in AMI patients has the greatest negative impact on the outcome of these patients during the first 30 days. This raises the question of whether earlier intervention in patients with significant AS leads to a better outcome of AMI patients. Our data provide a message that significant AS is not only independently associated with more severe heart failure and greater need for mechanical ventilation, but is also associated with mortality risk even after adjustment for possible covariates. Therefore, these patients must be treated with extreme caution.

The worse prognosis and survival of patients with AS is caused by the underlying pathophysiological mechanisms of AS, which result in oxygen demand-supply mismatch and ischemia, but also by the comorbidities and older age of these patients. Moreover, the acute ischemic event itself significantly enhances the progression of AS and MR. The worsening of AS severity develops more rapidly shortly after AMI, and a decrease in the rate of AS progression has been observed in the later period of a few years after AMI^13^ While pre-existing VHD is a significant factor contributing to adverse outcomes in these patients, the co-occurrence of AS and CAD often increases with age. As patients age, the prevalence of other comorbidities such as CKD and DM also increase, further complicating prognosis and impacting patient outcomes. The presence of these additional risk factors, along with left-sided VHD, should be carefully considered when assessing prognosis and planning treatment strategies. Given the complexity of managing patients with both pre-existing left-sided VHD and multiple comorbidities, a multidisciplinary approach is critical. Coordination between cardiologists, nephrologists, endocrinologists, and other healthcare providers is essential to address the full spectrum of patient needs, optimize treatment, and improve clinical outcomes.

Interestingly, according to our data, MR did not significantly increase the short-term mortality in multivariate analysis, but remained independently associated with long-term mortality even after adjustment for covariates. The progression of VHD after AMI may be explained by the post-AMI valvular remodeling caused by the neurohumoral activation, including the renin-angiotensin-aldosterone system, activation of fibrotic pathways, and increased collagen production and valve thickness^13,16,17^ Therefore, it is important to assess the progression of VHD severity and the need for valve intervention even shortly after MI and to continue in long-term follow-up. These patients need to be followed in hospitals with Heart teams and with facilities for surgical and transcatheter valve interventions.

## Conclusion

Preexisting significant VHD modifies the risk profile, presentation, and prognosis of patients with AMI. Patients with AS have the worst prognosis, which may indicate the need for earlier planning of valvular intervention, especially in those with current coronary disease.

## Data Availability

The data that support the findings of this study are not openly available due to reasons of sensitivity and are available from the corresponding author upon reasonable request.

## Abbreviations

AMI: Acute myocardial infarction
AS: Aortic stenosis
CAD: Coronary artery disease
LVEF: Left ventricular ejection fraction
MR: Mitral regurgitation
TIMI: Thrombolysis in Myocardial Infarction
VHD: Valvular heart disease
