# Publisher Correction: The impact of pre-existing aortic stenosis and mitral regurgitation on patients with acute myocardial infarction

**DOI:** 10.1038/s41598-025-14530-x

**Published:** 2025-08-07

**Authors:** Tamilla Muzafarova, Zuzana Motovska, Petr Kala, Ota Hlinomaz, Milan Hromadka, Teodora Vichova, Jan Mrozek, Marek Šramko, Martin Hutyra, Robert Petr, Pavol Tomasov, Oana Ionita, Pilar Lopez Santi, Aileen Paula Chua, Jiri Jarkovsky

**Affiliations:** 1 Cardiocentre, Third Faculty of Medicine, Charles University and University Hospital Kralovske Vinohrady, Srobarova 50, Prague, 10034 Czech Republic; 2 Department of Internal Medicine and Cardiology, Faculty of Medicine of Masaryk University and University Hospital Brno, Brno, Czech Republic; 3 First Department of Internal Medicine - Cardioangiology ICRC Faculty of Medicine of Masaryk, University and St. Anne’s University Hospital, Brno, Czech Republic; 4 Department of Cardiology, Faculty of Medicine in Pilsen, University Hospital, Charles University, Pilsen, Czech Republic; 5 Cardiovascular Department University Hospital Ostrava, Ostrava, Czech Republic; 6 Department of Cardiology Institute for Clinical and Experimental Medicine, Olomouc, Czech Republic; 7 First Internal, Cardiology Clinic University Hospital Olomouc, Olomouc, Czech Republic; 8 Cardiology Prague Ltd, Prague, Czech Republic; 9 Liberec Regional Hospital, Liberec, Czech Republic; 10 Leiden University Medical Centre, Leiden, Netherlands; 11 Institute of Biostatistics and Analyses Faculty of Medicine, Masaryk University, Brno, Czech Republic; 12 The Institute of Health Information and Statistics of the Czech Republic, Prague, Czech Republic

Correction to: *Scientific Reports* 10.1038/s41598-025-01313-7, published online 20 May 2025

The original version of this Article contained an error in the order of the author names, which was incorrectly given as Zuzana Motovska, Tamilla Muzafarova, Petr Kala, Ota Hlinomaz, Milan Hromadka, Teodora Vichova, Jan Mrozek, Marek Šramko, Martin Hutyra, Robert Petr, Pavol Tomasov, Oana Ionita, Pilar Lopez Santi, Aileen Paula Chua and Jiri Jarkovsky.

The original Article has been corrected.

